# Microglia Enhance Synapse Activity to Promote Local Network Synchronization

**DOI:** 10.1523/ENEURO.0088-18.2018

**Published:** 2018-10-25

**Authors:** Ryohei Akiyoshi, Hiroaki Wake, Daisuke Kato, Hiroshi Horiuchi, Riho Ono, Ako Ikegami, Koichiro Haruwaka, Toshiaki Omori, Yoshihisa Tachibana, Andrew J. Moorhouse, Junichi Nabekura

**Affiliations:** 1 Division of Homeostatic Development, National Institute for Physiological Sciences, National Institutes of Natural Sciences, Okazaki, Japan 444-8585; 2 Department of Physiological Sciences, the Graduate School for Advanced Study, Hayama, Japan 240-0193; 3 Division of System Neuroscience, Kobe University Graduate School of Medicine, Kobe, Japan 650-0017; 4 Department of Electrical and Electronic Engineering, Graduate School of Engineering, Kobe University, Kobe, Japan 657-8501; 5 School of Medical Sciences, University of New South Wales, Sydney, Australia 2052; 6 Core Research for Evolutional Science and Technology, Japan Science and Technology Agency, Saitama, Japan 100-0004; 7 Precursory Research for Embryonic Science and Technology, Japan Science and Technology Agency, Saitama, Japan 100-0004

**Keywords:** calcium imaging, in vivo, microglia, synapse, two photon

## Abstract

Microglia are highly motile immunoreactive cells that play integral roles in the response to brain infection and damage, and in the progression of various neurological diseases. During development, microglia also help sculpt neural circuits, via both promoting synapse formation and by targeting specific synapses for elimination and phagocytosis. Microglia are also active surveyors of neural circuits in the mature, healthy brain, although the functional consequences of such microglia-neuron contacts under these conditions is unclear. Using *in vivo* imaging of neurons and microglia in awake mice, we report here the functional consequences of microglia-synapse contacts. Direct contact between a microglial process and a single synapse results in a specific increase in the activity of that contacted synapse, and a corresponding increase in back-propagating action potentials along the parent dendrite. This increase in activity is not seen for microglia-synapse contacts when microglia are activated by chronic lipopolysaccharide (LPS) treatment. To probe how this microglia-synapse contact affects neural circuits, we imaged across larger populations of motor cortical neurons. When microglia were again activated by LPS (or partially ablated), there was a decrease in the extent to which neuronal activity was synchronized. Together, our results demonstrate that interactions between physiological or resting microglia and synapses in the mature, healthy brain leads to an increase in neuronal activity and thereby helps to synchronize local populations of neurons. Our novel findings provide a plausible physical basis for understanding how alterations in immune status may impact on neural circuit plasticity and on cognitive behaviors such as learning.

## Visual Abstract

**Figure:**
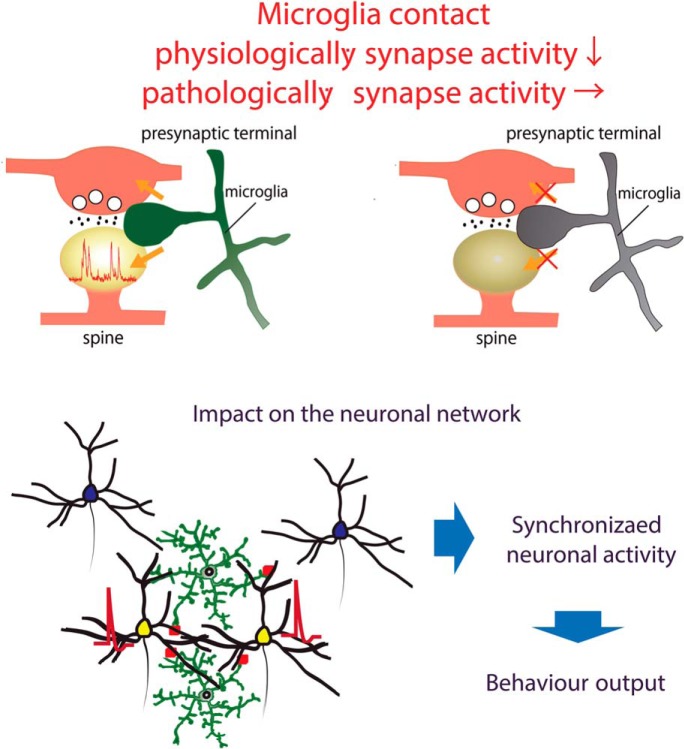

## Significance Statement

Microglia, the sole immune cells in the central nervous system, make frequent contacts with synapses on dendritic spines, but the functional significance of these contact has remained elusive. In this study, we use *in vivo* two photon imaging and demonstrate that microglia contact on spines increases synaptic activity. This microglia-induced increase in synaptic activity enhances the synchronization of neuronal populations. This increase synchrony is inhibited by microglial activation, proving a possible mechanistic basis for how immune status may impact on neural circuit function.

## Introduction

Microglia are highly motile immune effector cells in the brain that respond to neuronal infection and damage by converting from a resting or physiologic phenotype, to an activated or reactive phenotype. This reactive phenotype is associated with morphologic changes, migration and proliferation, release of inflammatory and neuroactive molecules, and ultimately phagocytosis of damaged neuronal elements (Kettenmann et al., 2011). Such microglia activation is a hallmark of the pathogenesis of neurodegenerative diseases such as Alzheimer’s disease, Parkinson’s disease, and amyotrophic lateral sclerosis (Cunningham, 2013). Whether microglia activation occurs early in the disease pathogenesis to trigger some aspects of neuronal dysfunction is less clear. A broader question is to what extent disruptions in the interactions between physiologic microglia and neural circuits, such as may occur in response to microglial activation, impacts on neuronal homeostasis and cognitive performance (Salter and Beggs, 2014; Estes and McAllister, 2015; Kipnis, 2016; Tay et al., 2017). These physiologic microglia are far from resting, they actively survey the brain parenchyma with their processes making frequent and direct contacts with neuronal synapses (Nimmerjahn et al., 2005; Wake et al., 2009). This contact between microglial processes and the various neuronal elements appears to occur in an activity dependent fashion (Dissing-Olesen et al., 2014; Eyo et al., 2014), but the consequences of this interaction for neural circuit homeostasis and plasticity in the mature, healthy brain are not fully understood. Microglia neuronal contacts can physically sculpt neural circuits, through phagocytosing weaker or inactive synapses during development and after injury (Schafer et al., 2012), and through promoting neuronal synapse and/or spine formation either directly or indirectly (Parkhurst et al., 2013; Miyamoto et al., 2016). However, the acute effects of microglia-neuron contacts on neural activity are less clear. In immature zebrafish neurons, microglia-neuron contacts can reduce neuronal activity (Li et al., 2012), and we proposed that interactions between physiologic microglia and neuronal synapses modulates neural circuit activity in the mature, healthy mammalian brain. To examine this hypothesis, we combined *in vivo* imaging of physiologic and activated microglia with imaging of neuronal activity in awake mice, at both the single synapse level and across neural circuits. Our results demonstrate that physiologic microglia can selectively enhance the activity of synapses and neurons that they contact. We show that this microglia-neuron contact results in an increase in the synchronization of activity across local neuronal populations. Our results have marked implications for the understanding of how immune status can impact on neural network activity and cognitive function, and suggest that microglia could potentially play a primary role in cognitive dysfunction associated with aging and psychiatric diseases.

## Materials and Methods

### Animals and microglia ablation or activation

All animal experiments were approved by the Animal Research Committees. Mice were given free access to food and water in a 12/12 h light/dark cycle, and we used male mice for all experiments. To image microglia, we used ionized Ca^2+^-binding adapter molecule 1 (Iba1)-enhanced green fluorescent protein (EGFP) transgenic mice, which expresses EGFP under the control of the Iba1 promoter, which is specific for microglia and macrophages (Hirasawa et al., 2005). For microglia ablation experiments, we crossed Iba1-tetracycline transactivator (Iba1-tTA) mice (Tanaka et al., 2012) with tetracycline operator-diphtheria toxin A (tetO-DTA) mice (Stanger et al., 2007). Withdrawal of doxycycline (Dox-off) in the feed of these mice leads to selective expression of the DTA in microglia. All transgenic mice were derived from the C57BL/6J strain. Mice were reared with standard chow containing Dox 0.1 g kg^−1^. This was replaced with Dox-free standard chow from seven weeks after birth to induce transgene induction, a time corresponding to 7 d before the first imaging experiment. Lipopolysaccharide (LPS; Funakoshi) was used to pharmacologically activate microglia, and was injected daily (1.0 mg kg^−1^, i.p.) for 9 d. Control mice received intraperitoneal saline injections with the same dosing schedule. Imaging was performed on day 4 and day 9 during the LPS or saline injection. In an additional cohort used to examine time course and recovery, chronic imaging was performed before LPS, and 1 and 4 d after daily LPS injections, and again at 7 d after stopping LPS.

### Surgery and adeno-associated virus (AAV) injection

All mice were anesthetized by ketamine (74 mg/kg, i.p.) and xylazine (10 mg/kg, i.p.). The skin was disinfected with 70% (w/vol) ethanol, the skull was exposed and cleaned, and a custom-made head plate was firmly attached using dental cement (Fujiryu-to BC; GC, Bistite II; Tokuyama Dental). The surface of the intact skull was subsequently coated with an acrylic-based dental adhesive resin cement (Super bond; Sun Medical) to avoid drying. Mice were allowed to recover for 1 d before open skull surgery and/or viral injection.

Under isoflurane (1%) anesthesia, a circular craniotomy (∼2 mm in diameter) was performed (Masamizu et al., 2014) over the left primary motor cortex (centered at 0.2 mm anterior and 1 mm lateral from bregma). To visualize neuronal activity in L2/3 pyramidal neurons, a rAAV2/1-Syn-GCaMP6 AAV (UPenn Vector Core; 9.0 × 10^12^ vector genomes/ml, diluted 1:5 in PBS) was injected at three sites in L2/3 of the cortex using a glass pipette (tip diameter: 10 μm), which was left in the brain for an additional 10 min to avoid backflow. For structural and Ca^2+^ imaging of apical dendrites and spines of layer 5 (L5) pyramidal neurons, a mixed AAV [UPenn Vector Core; AAV2/1-Syn-FLEX-GCaMP6: 1.09 × 10^13^ vector genomes/ml, AAV2/1-CAG-FLEX-Tdtomato: 7.6 × 10^12^ vector genomes/ml, and AAV2/1-CaMKII -Cre: 2.94 × 10^13^ vector genomes/ml (1: 10 000) diluted in PBS, three sites] solution was similarly injected into L5 of the cortex. After the injection, 2% (w/v) agarose L (Nippon Gene) was placed over the brain surface and a glass window comprised of two coverslips (Matsunami Glass) sealed with an ultraviolet curable adhesive (NOR-61, Norland) was implanted over the craniotomy. The edges of the cranial window were sealed with dental cement and dental adhesive resin cement.

### Two-photon imaging

Ca^2+^ imaging of L2/3 pyramidal neurons was conducted using a LSM 7 MP system (Zeiss) and a mode-locked Ti:sapphire Chameleon Ultra II laser (Coherent) tuned to 950 nm, with a 20× objective (XLPlan, NA of 1.0; Zeiss). Fluorescence was separated by a 570-nm dichroic mirror with 495–550 nm (green channel: for EGFP fluorescence detection) and 570–630 nm (red channel: for tdTomato fluorescence detection) emission filters and was collected using GaAsP photomultiplier tubes (Hamamatsu Photonics). The laser intensity was 5–30 mW. The imaged fields were 424.27 × 424.27 μm, at a depth of 150–200 μm below the cortical surface. The pixel size was 0.830 μm. Frame duration was 390 ms, and continuous 1000-frame imaging was repeated for each field for 10–30 min. For imaging the apical dendrites (and their spines) of L5 pyramidal neurons in awake mice, the image plane was focused at a depth within 50 μm from the cortical surface (L1). The image fields were 28.28 × 28.28 μm, with a pixel size of 0.055 μm and a frame duration of 242 ms. Continuous 3000-frame imaging was repeated in each field for 12 min.

### Image analysis

Analyses were performed using an ImageJ plug-in (1.37v; NIH) and programs written in MATLAB (version 8; MathWorks). Movies were corrected for focal plane displacements using TurboReg (Thevenaz et al., 1998). To quantify neural activity, regions of interest (ROIs) around a cell were determined using a semi-automated algorithm that correlated the fluorescence intensity between adjacent pixels to define the cell edge, and ROIs were confirmed by visual inspection. Note this procedure only detects neurons with fluorescent responses. For time lapse imaging of interactions between microglia and neurons, ROIs were manually defined around single spines or around dendrites. The fluorescence within a ROI was quantified for each image and averaged over time, with background fluorescence subsequently subtracted from this averaged fluorescence. Slow timescale changes in fluorescence were removed from the fluorescence time series values using the method of (Dombeck et al., 2007), in which the distribution of fluorescence in a ±15-s interval around each sample time point was determined, and the 8th percentile was subtracted. For identification of significant transients in each ROI, the baseline and SD (σ) were calculated from manually selected large transients-free regions. A fluorescent Ca^2+^ transient was detected if the fluorescent intensity (FI) exceeded 4 SDs (σ) above the mean baseline. The correlation coefficient (C.C.) was obtained by quantifying the time in which the Ca^2+^ transient was at its peak value, and grouping these times into 242-ms bins.

ImageJ was used to quantify contacts between GFP-labeled microglia and tdTomato-labeled spines. Images were manually screened across all time points and across different Z planes to detect contacts as overlap between the red (spine) and the green (microglia) fluorescent channels, after determining the baseline thresholds for each channel respectively. Real contacts were defined only if the red and the green fluorescence overlap occurred in at least two Z sections.

### Immunohistochemistry

To quantify microglia morphology, mice were transcardially perfused with 4% paraformaldehyde in phosphate buffer (PB; pH 7.4), and brains were excised and fixed overnight in the same solution. Fixed brains were equilibrated in a 30% sucrose solution in PB, and cut with a microtome (Leica Microsystems) into 30-μm-thick sections. Sections containing primary motor cortex were incubated overnight with primary antibody (anti-Iba1 antibody, 1:500 diluted in 10% normal goat serum/PBST; Wako) at 4°C, followed by 1-h incubation with secondary antibody (Alexa Fluor 488-conjugated donkey anti-mouse; Invitrogen). Fluorescent signals were detected with a LSM 5 DUO system (Zeiss).

### Image analysis for microglia morphology

Iba1-labeled fixed brain slices were imaged on a confocal microscope with an Argon laser operating at a wavelength of 488 nm. A laser scanning system (Carl Zeiss MicroImaging, Inc.) with water-immersion objective (20×, 1.0 NA, Zeiss) was used for image acquisition. Z stack images (512 × 512 pixels, 0.198-μm pixel-1, 0.5-μm Z-step) were used for morphologic analysis of microglia. The extent of ramification of microglia processes was quantified by circumscribing the area of the distal portions of microglial processes using the segmented line tool in ImageJ. Microglia size was measured by circumscribing the cell body using an intensity threshold tool in ImageJ. Morphometric quantification was done with Neurolucida, performing a Sholl analysis of process number and length with respect to standard distances from the soma.

### Data and statistics

Data are presented as mean ± SEM. Unpaired, paired, and one-sample *t* test, Wilcoxon rank sum tests, and Pearson’s correlation tests were used to test for statistical significance as indicated. When analyzing the Ca^2+^ imaging data in both soma and spines, we first performed the D’Agostino and Pearson normality test, which indicated data were not normally distributed.

## Results

### Physiologic microglial contact with synapses enhances neuronal activity

**Figure 1.**
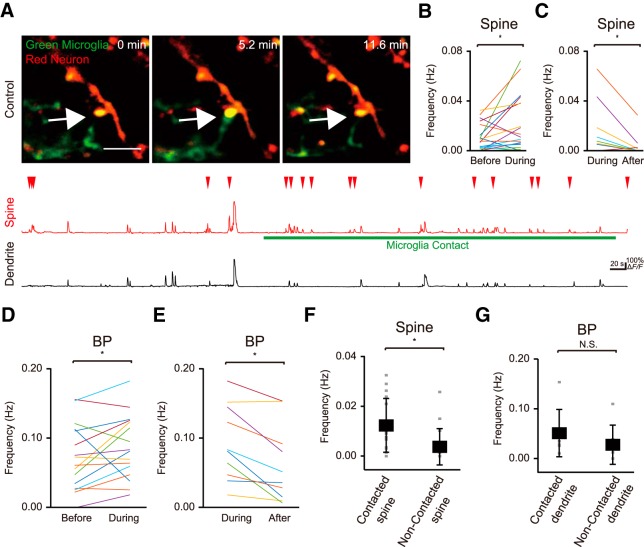
Enhanced synapse activity follows microglia contact. ***A***, top, Fluorescent image of a dendrite in in L2/3 of the primary motor cortex showing the simultaneous imaging of spines and their parent dendrite (tdTomato; red) and microglia (EGFP, green). Scale bar: 5 μm. The image panels show microglial process contacting a spine (center). Lower traces, Activity in the neuronal compartments was measured with GCaMP6f and quantified as Δ*F/F.* Sample Ca^2+^ traces from a spine and its parent dendrite before, during, and after microglia contact are shown. Putative backpropagating action potentials cause synchronous responses in both dendrite and spine, whereas presumed local synaptic activity results in spine transients only. Note the enhanced local (spine) synaptic activity with microglial contact. The white arrow indicate the spine contacted by microglia process. ***B***, ***C***, The frequency of local spine Ca^2+^ transients were increased during microglia contact periods relative to those before (***B***) or after (***C***) contact [***B***: 0.020 ± 0.005 Hz (during) vs 0.010 ± 0.002 Hz (before), *p* = 0.033, *n* = 20 fields from 17 mice; ***C***: 0.016 ± 0.007 Hz (during) vs 0.004 ± 0.003 Hz (after), *p* = 0.030, *n* = 10 fields from 10 mice, paired *t* test]. ***D***, ***E***, The frequency of back propagating action potentials reflected as dendritic Ca^2+^ transients was increased during the period of microglia-synapse contact, relative to that before (***D***) or after (***E***) contact [***D***: 0.089 ± 0.011 Hz (during) vs 0.074 ± 0.012 Hz (before), *p* = 0.044, *n* = 20 fields from 17 mice; ***E***: 0.093 ± 0.017 Hz (during) vs 0.063 ± 0.017 Hz (after), *p* = 0.003, *n* = 10 fields from 10 mice, paired *t* test]. ***F***, Microglia were attracted to synapses with a higher basal (pre-contact) frequency of local Ca^2+^ responses (spines with microglia contact: 0.012 ± 0.011 Hz; spines without microglia contact: 0.004 ± 0.007 Hz, *p* = 0.004, *n* = 16 fields from 15 mice, Wilcoxon rank sum test). ***G***, In contrast, contact frequency did not depend on the basal frequency of back propagating action potentials (dendrites with microglia contact: 0.047 ± 0.048 Hz; dendrites without microglia contact: 0.028 ± 0.040 Hz, *p* = 0.32, *n* = 7 fields from seven mice, Wilcoxon rank sum test). Data are presented as the mean ± SEM; *p < 0.05.

In the healthy, mature cortex, quiescent or physiologic microglial processes are highly motile and survey the neuronal environment (Nimmerjahn et al., 2005; Wake et al., 2009). These motile processes specifically and directly contact synapses, and the frequency and extent of these contacts depends on neuronal activity (Wake et al., 2009; Tremblay et al., 2010; Li et al., 2012). To probe the functional consequences of these microglia-synapse contacts, we simultaneously visualized neuronal activity and microglial processes, using time-lapse *in vivo* imaging in awake mice. Microglia were identified by EGFP fluorescence driven by a microglia-specific promoter (using Iba1-EGFP mice), while neuronal activity was monitored using injection of an AAV expressing the Ca^2+^ sensor, GCaMP6f, along with the reporter construct tdTomato (Fig. 1*A*). As we reported previously (Wake et al., 2009), microglial processes made direct and brief (≈5 min) contacts with synapses located on dendritic spines (Fig. 1*A*). Using high resolution imaging we could detect and quantify localized Ca^2+^ transients within single dendritic spines, during which time (up to 12 min) we sometimes observed a period of microglial contact. During the microglial contact period, the frequency of spine Ca^2+^ transients was significantly increased as compared to that either before or after contact [Fig. 1*A–C*; 0.020 ± 0.005 Hz (during) vs 0.010 ± 0.002 Hz (before), *p* = 0.033, *n* = 20 fields from 17 mice (Fig. 1*B*); 0.016 ± 0.007 Hz (during) vs 0.004 ± 0.003 Hz (after), *p* = 0.030, *n* = 10 fields from 10 mice (Fig. 1*C*), paired *t* test]. The increase in spine activity was consistently seen, although variable in extent, and was clearly reversible on withdrawal of the microglial process from the spine (Fig. 1*A–C*).

Ca^2+^ transients occurring along the entire dendrite, and that were co-incident with dendritic spine transients, were also observed in these experiments, presumably reflecting back-propagating action potentials (BPs) originating from the neuronal soma. The frequency of these BP-associated dendritic Ca^2+^ transients was also increased during microglia contact onto synapses [Fig. 1*D*,*E*; 0.089 ± 0.011 Hz (during) vs 0.074 ± 0.012 Hz (before), *p* = 0.044, *n* = 20 fields from 17 mice (Fig. 1*D*); 0.093 ± 0.017 Hz (during) vs 0.063 ± 0.017 Hz (after), *p* = 0.003, *n* = 10 fields from 10 mice (Fig. 1*E*), paired *t* test], suggesting the increased spine activity associated with microglia contact also resulted in an increase in excitability of the neuron at the soma (Fig. 1*D*,*E*).

To examine how microglial processes may select particular spines to be contacted, we compared (in different experiments from above) the basal rate of Ca^2+^ transients in spines and dendrites in contacted and non-contacted spines within the same image fields (Fig. 1*F*). Those spines that were contacted by microglia had significantly higher basal rates of Ca^2+^ transients before the microglia contacts (Fig. 1*F*; 0.012 ± 0.011 Hz) compared to spines without any apparent contacts (Fig. 1*F*; 0.004 ± 0.007 Hz, *p* = 0.004, *n* = 16 fields from 15 mice, Wilcoxon rank sum test). However, this higher basal rate was restricted to the spines, as the frequency of Ca^2+^ transients in the parent dendrite before microglia contact was not different from that in dendrites with spines that were not-contacted (Fig. 1*G*; dendrites with microglia contact; 0.047 ± 0.048 Hz, dendrites without microglia contact; 0.028 ± 0.040 Hz, *p* = 0.32, *n* = 7 fields from seven mice, Wilcoxon rank sum test). Together, this suggests a very localized level of neuronal activity restricted just to the synapse influences microglial process contact. Such local activity may evoke an attractant signal with minimal spatial influence, as has been suggested previously (Wake et al., 2009; Dissing-Olesen et al., 2014).

**Figure 2.**
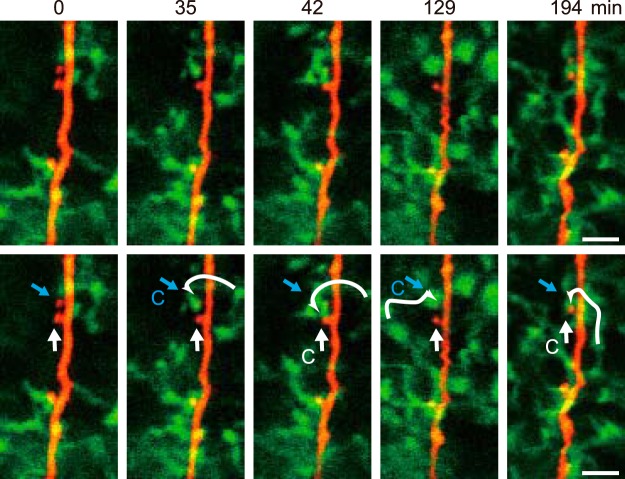
Specific physiologic microglial processes contact different spines while a single spine may be contacted by different microglia. Representative long-term simultaneous images of microglia (green) and of dendrites and spines (red), showing examples of microglial-synapse contacts during over three consecutive hours. The upper and lower panels show the same images with the lower panels annotated by white and blue straight arrows to indicate two different spines, and with a curved white arrow to indicate the trajectory of for a single microglial process as it contacts a spine (contact indicated by C). The upper spine (blue arrow) is contacted by one microglia at 35–42 min, and by a seemingly different microglia at 129 min. At 42 and 194 min, the original microglia contacts the lower (white arrow) spine.

After retracting from a dendritic spine, a microglial process continued surveying the local environment and could make a second contact with a different spine on the same dendrite (Fig. 2). This is consistent with microglia being attracted to more active synapses and with the localized region of surveillance of each microglia. However, the same spine could also be again contacted by a different microglial process (Fig. 2). As the frequency of contacts within the same image field was only about once per hour, subsequent longer-term imaging will be needed to quantify the patterns of contact by each single microglia.

### The physiologic microglial phenotype is necessary for synchrony of local network activity

**Figure 3.**
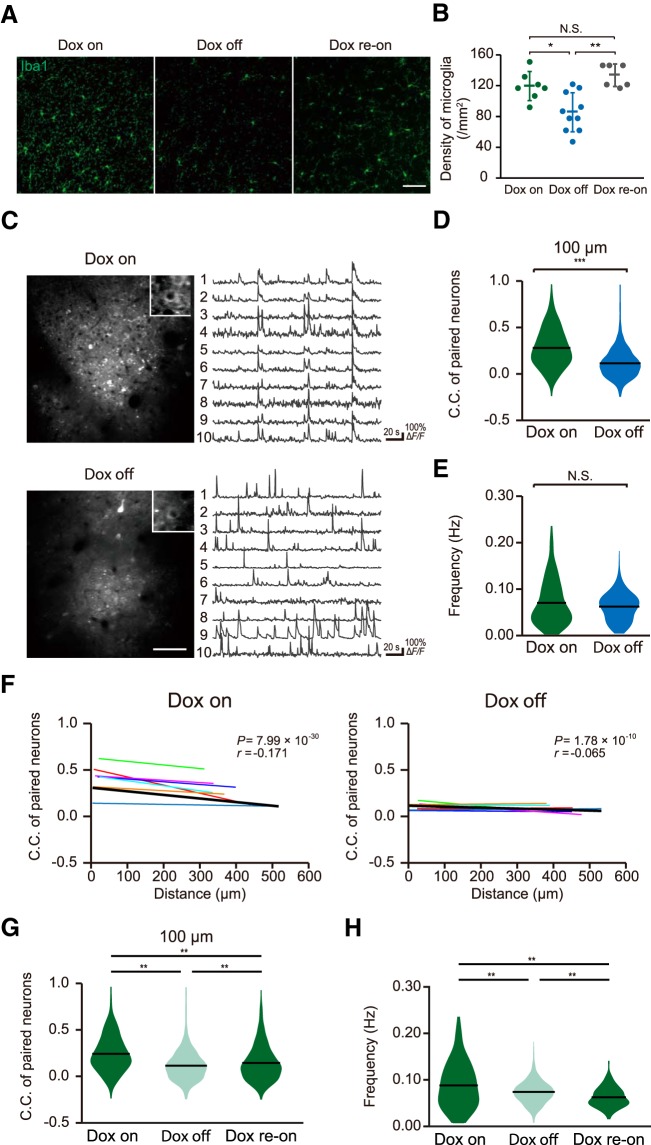
Decreased synchronization of evoked neural activity in microglia ablated mice. ***A***, Fluorescent (immunohistochemistry) images of Iba1-positive microglia in cortical sections of Dox on (left), Dox off (center), and re-Dox on mice showing reduced density of Iba1 (microglia)-positive cells in Iba1-tTA::tetO-DTA transgenic mice following 7 d of Dox withdrawal as compared with Iba1-tTA::tetO-DTA transgenic mice with Dox maintained in the diet and in Dox-off mice after Dox had been returned to the diet for a week (Dox re-on). Scale bar in right panel: 50 μm. ***B***, Genetic ablation of microglia was verified by quantification of microglia density. Dox-off mice showed a significant reduction in the density of microglia from 121.3 ± 18.7/mm^2^ (*n* = 7 fields from three Dox on mice) to 87.4 ± 25.1/mm^2^ (*n* = 10 fields from three Dox-off mice; *p* = 0.006, unpaired *t* test). The density of microglia recovered to control levels following one week of Dox return to the diet (135.0 ± 14.5/mm^2^, *n* = 6 fields from three Dox re-on mice; *p* = 0.0008, unpaired *t* test). ***C***, left, Typical fields of view of L2/3 primary motor cortex neurons expressing GCaMP6f in Dox on mice (upper) and Dox off mice (lower). Right, Representative Δ*F/F* Ca^2+^ traces from individual GCaMP6f-positive neurons from Dox on (upper) and Dox off mice (lower). ***D***, Synchronous neuronal firing was estimated by measuring the proportion of neuron pairs in which Ca^2+^ transients were seen at the same time, and quantifying this as a C.C. The mean C.C. of paired neurons within 100 μm of each other was lower in Dox off mice as compared with that in Dox on mice [Dox on mice (*n* = seven mice); 0.28 ± 0.006, Dox off mice (*n* = 7 mice); 0.12 ± 0.003, *p* = 1.75 × 10^−171^, Wilcoxon rank sum test]. ***E***, There was no difference in the frequency of Ca^2+^ responses between Dox on and Dox off mice [Dox on (*n* = 269 neurons from seven mice); 0.071 ± 0.003 Hz, Dox off (*n* = 326 neurons from seven mice); 0.062 ± 0.002 Hz, *p* = 0.463, Wilcoxon rank sum test]. ***F***, The C.C.s in each pair of neurons was negatively correlated with the distance separating each pair of neurons in control (Dox on) mice (left). However, this negative correlation was absent in Dox off mice (right). Individual slope correlations from each mouse are shown by the thin colored regression lines, while the thicker black line shows the correlation obtained by fitting all the data simultaneously. The value of the slope of this group correlation and the *p* value obtained from the Pearson’s correlation test are shown in the top right of each graph, while averaged slope values obtained from each mouse are shown in this legend (control mice: *n* = 7, *r* = –0.171, *p* = 7.99 × 10^−30^; microglia ablated mice: *n* = 7, *r* = –0.065, *p* = 1.78 × 10^−8^, Pearson’s correlation test; for seven Dox on mice, *r* = –0.175 ± 0.045, *p* = 0.0082, one-sample *t* test; for seven Dox off mice, *r* = –0.048 ± 0.036, *p* = 0.227, one-sample *t* test). ***G***, ***H***, In an additional cohort of mice, the C.C.s of neurons located within 100 μm of each other (***G***) and the neuron firing frequencies (***H***) were measured during the doc on diet (left), following 7 d of Dox off diet (center) and again 42 d after returning to dox diet (dox re-on, right). Dox-off again reduced the C.C. which partially recovered after returning to dox [***G***: *F*_(2,4926)_ = 200.21, *p* = 9.56 × 10^−10^ (Dox on: 0.243 ± 0.006 vs Dox off: 0.115 ± 0.003), *p* = 3.15 × 10^−7^ (Dox off: 0.115 ± 0.003 vs Dox re-on: 0.145 ± 0.005), *p* = 9.56 × 10^−10^ (Dox on: 0.243 ± 0.006 vs Dox re-on: 0.145 ± 0.005), one-way ANOVA followed by Tukey’s test]. The neuronal firing frequencies in this cohort were decreased after Dox off and decreased further after returning to dox on [***H***: Dox on (*n* = 165 neurons, four fields from two mice); 0.088 ± 0.004 Hz, Dox off (*n* = 229 neurons, four fields from two mice); 0.074 ± 0.001 Hz, Dox re-on (*n* = 211 neurons, four fields from two mice); 0.063 ± 0.001 Hz, *F*_(2,602)_ = 29.19, *p* = 5.83 × 10^−5^ (Dox on vs Dox off), *p* = 5.15 × 10^−4^ (Dox off vs Dox re-on), *p* = 9.56 × 10^−10^ (Dox on vs Dox re-on), one-way ANOVA followed by Tukey’s test]. Data in panel ***B*** are presented as the mean ± SEM, while data in panels ***D***, ***E***, ***G***, ***H*** show means and distributions of each data point; *p < 0.05, ***p < 0.001, n.s.: non-significant.

To examine how microglial contact-induced synapse modulation may affect neuronal circuit activity, we imaged activity in larger populations of neurons and compared results with mice with reduced microglia population. Ablation of microglia was achieved by crossing Iba1-tTA mice (Tanaka et al., 2012) with TetO-DTA mice (Stanger et al., 2007) to establish an Iba1-tTA-TetO-DTA mouse colony. Dox-off from the chow of these mice causes selective expression of the DTA in microglia, resulting in a marked reduction in the microglia population. It has previously been shown that the levels of cytokines are not elevated with microglia ablation (Parkhurst et al., 2013). As expected, dox removal for 7 d resulted in a significant reduction in the density of microglia (Fig. 3*A*,*B*) from 121.3 ± 18.7/mm^2^ (*n* = 7 fields from three Dox on mice) to 87.4 ± 25.1/mm^2^ (*n* = 10 fields from three Dox-off mice; *p* = 0.006, unpaired *t* test). To quantify neural circuit activity, we injected the GCaMP6f expressing AAV into these mice and quantified Ca^2+^ fluorescence across multiple neurons in L2/3 of the primary motor cortex (Fig. 3*C–F*). As shown in Figure 3*E*, the frequency of Ca^2+^ activity in L2/3 neurons from Dox on control mice was not significantly different from that in Dox off mice [Fig. 3*E*; Dox on (*n* = 269 neurons from seven mice); 0.071 ± 0.003 Hz, Dox off (*n* = 326 neurons from seven mice); 0.062 ± 0.002 Hz, *p* = 0.463, Wilcoxon rank sum test]. To quantify neuronal synchronization, we defined a C.C. between every individual pair of neurons within the image area in each mouse. The C.C. of activity in paired neurons in microglia ablated mice (Dox off for 7 d) was reduced compared to control (Dox on) littermates [Fig. 3*D*; control mice (*n* = seven mice); 0.28 ± 0.006, microglia ablated mice (*n* = 7 mice); 0.12 ± 0.003, *p* = 1.75 × 10^−171^, Wilcoxon rank sum test]. Previous work in the M1 motor cortex has shown that correlations between activity of paired neurons varies as a function of the distance separating the neurons (Hira et al., 2013), with neurons closer together more likely to fire together. Hence, we plotted the C.C. against the distance between each of the neurons within the pair to examine if this relationship is changed in microglia ablated mice (Fig. 3*F*). In control mice, there was a weak inverse relationship between C.C. and separation distance (*r* = –0.171, *p* = 7.99 × 10^−30^, Pearson’s correlation test), indicating that L2/3 neurons located closer to each other are more likely to fire together. In contrast, this correlation was diminished in microglia ablated mice (*r* = –0.065, *p* = 1.78 × 10^−8^, Pearson’s correlation test). To further validate this difference, we averaged the slope of the relationship between C.C. and neuronal separation distance in each mouse within the Dox on and off groups. The relationship between C.C. and neuronal separation was abolished by microglial ablation (Fig. 3*F*; Dox on: *n* = 7, *r* = –0.175 ± 0.045, *p* = 0.0082, Dox off: *n* = 7, *r* = –0.048 ± 0.036, *p* = 0.227, one-sample *t* test). Together, our population imaging indicates that microglia ablation results in reduced synchronous firing of L2/3 neurons, and particularly for neurons located close to each other. To test whether the reduction of C.C. on microglial recovery recovered once the microglial population was restored, we conducted an additional experimental cohort where we tracked neuronal activity and C.C. in the same mice before Dox-off, after 7 d Dox off, and again after returning the mice to Dox-on chow for 42 d. The density of microglia completely recovered with even just one week of Dox re-administrating [135.0 ± 14.5/mm^2^ (*n* = 6 fields from three mice); Fig. 3*A*,*B*]. As shown in Figure 3*G*,*H*, there was again a significant reduction of C.C. on Dox-off, which partially recovered following Dox return [Fig. 3*G*; *F*_(2,4926)_ = 200.21, *p* = 9.56 × 10^−10^ (Dox on: 0.243 ± 0.006 vs Dox off: 0.115 ± 0.003), *p* = 3.15 × 10^−7^ (Dox off: 0.115 ± 0.003 vs Dox re-on: 0.145 ± 0.005), *p* = 9.56 × 10^−10^ (Dox on: 0.243 ± 0.006 vs Dox re-on: 0.145 ± 0.005), one-way ANOVA followed by Tukey’s test]. In this cohort, there was also a modest (but significant) decrease in neuronal firing frequency after Dox off, which further decreased slightly after 42 d of Dox return [Fig. 3*H*; Dox on (*n* = 165 neurons, four fields from two mice); 0.088 ± 0.004 Hz, Dox off (*n* = 229 neurons, four fields from two mice); 0.074 ± 0.001 Hz, Dox re-on (*n* = 211 neurons, four fields from two mice); 0.063 ± 0.001 Hz, *F*_(2,602)_ = 29.19, *p* = 5.83 × 10^−5^ (Dox on vs Dox off), *p* = 5.15 × 10^−4^ (Dox off vs Dox re-on), *p* = 9.56 × 10^−10^ (Dox on vs Dox re-on), one-way ANOVA followed by Tukey’s test]. Hence, partial microglia ablation results in an apparent irreversible reduction in synchronicity, which we propose results from reduced microglia-synapse contact induced modulation of neural activity.

### The impact of microglial-synapse contact on neuronal activity is lost on microglial activation

**Figure 4.**
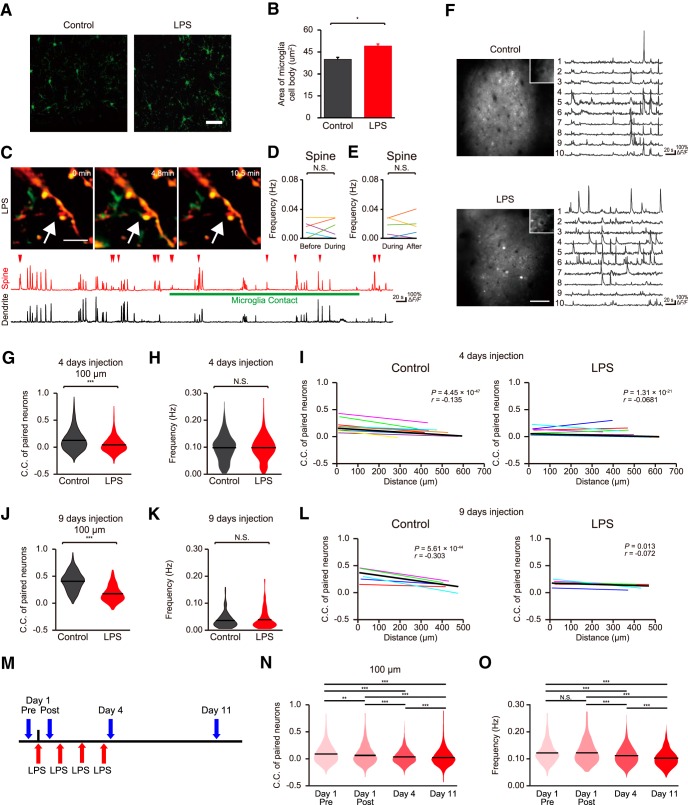
Microglia-synapse interactions in mice following LPS injections. ***A***, ***B***, Intraperitoneal injection of LPS activated microglia, as verified by morphologic appearance (***A***), which included a significant increase in soma diameter (***B***). Soma area increased from 40.1 ± 9.2 μm^2^ in saline-injected mice (*n* = 58 cells from three mice) to 49.2 ± 11 μm^2^ in LPS-injected mice (*n* = 80 cells from three mice, *p* = 4.30 × 10^−7^, unpaired *t* test). ***C***, top, Fluorescent image of a dendrite in L2/3 of the primary motor cortex of an LPS-injected mice showing the simultaneous imaging of spines (tdTomato; red) and microglia (EGFP; green). Scale bar: 5 μm. As in Figure 1, the lower trace shows a sample recording of Ca^2+^ transients (GCaMP6f fluorescence Δ*F/F*) in a single spine (upper) and in the parent dendrite (lower) before, during, and after microglial contact. Local synaptic activity was not effected by microglial contact. The white arrow indicate the spine contacted by microglia process. ***D***, ***E***, The frequency of Ca^2+^ transients in spines during the period of microglial contact was not significantly different from that either before (***D***) or after (***E***) contact. Each line shows data from a single experiment [***D***: 0.013 ± 0.003 Hz (during) vs 0.012 ± 0.003 Hz (before), *p* = 0.816, *n* = 6 fields from three mice; ***E***: 0.013 ± 0.004 Hz (during) vs 0.014 ± 0.005 Hz (after), *p* = 0.843, *n* = 6 fields from three mice, paired *t* test]. ***F***, Typical fields of view of neurons with GCaMP6f fluorescence in L2/3 of the primary motor cortex of saline-injected mice (upper) and LPS-injected mice (lower), with accompanying representative Δ*F/F* Ca^2+^ traces from ten individual neurons in each mouse. ***G–L***, Distribution of C.C.s (***G***, ***J***) and of neuronal firing frequencies (***H***, ***K***) in mice injected with either saline or LPS for 4 d (***G***, ***H***) or 9 d (***J***, ***K***). LPS injections decreased the C.C. for pairs of closely located neurons but had no effect on neuronal firing frequencies. Panels ***I***, ***L*** show the relationship between C.C. and neuronal separation for mice injected with either saline or LPS for 4 d (***I***) or 9 d (***L***). Each line shows the slope of the correlation from individual mice, while the values in the top right corner show the slope of the fits to the complete data set (and the value of the Pearson’s correlation test), the fit to this grouped data are shown as the solid dark line. Averaging the individual fits gives a mean slope correlation of: *r* = –0.149 ± 0.019 for 4 d saline mice (*p* = 5.43 × 10^−5^, one-sample *t* test, *n* = 9 mice), *r* = –0.008 ± 0.039 for 4 d LPS mice (*p* = 0.850, *n* = 9 mice), *r* = –0.285 ± 0.065, for 9 d saline (*p* = 0.012, *n* = 5 mice), and *r* = –0.107 ± 0.048 for 9 d LPS [*p* = 0.091, *n* = 5 mice; ***G***: saline-injected mice (4 d injection, *n* = 9 mice); 0.12 ± 0.004, LPS-injected mice (4 d injection, *n* = 9 mice); 0.043 ± 0.002, *p* = 7.02 × 10^−72^, Wilcoxon rank sum test; ***H***: control (4 d injection, *n* = 464 neurons from nine mice); 0.098 ± 0.002 Hz, LPS (4 d injection, *n* = 568 neurons from nine mice); 0.098 ± 0.002 Hz, *p* = 0.841, Wilcoxon rank sum test; ***I***; control (4 d injection); *r* = –0.135, *p* = 4.45 × 10^−47^, LPS (4 d injection); *r* = –0.0681, *p* = 1.31 × 10^−21^, Pearson’s correlation test; ***J***; saline-injected mice (9 d injection, *n* = 5 mice); 0.34 ± 0.008, LPS-injected mice (9 d injection, *n* = 5 mice); 0.18 ± 0.009, *p* = 3.61 × 10^−30^, Wilcoxon rank sum test; ***K***; saline-injected mice (9 d injection, *n* = 141 neurons from five mice); 0.033 ± 0.002 Hz, LPS-injected mice (9 d injection, *n* = 108 neurons from five mice); 0.037 ± 0.003 Hz, *p* = 0.60, Wilcoxon rank sum test; ***L***; control (9 d injection); *r* = –0.303, *p* = 5.61 × 10^−44^, LPS (9 d injection); *r* = –0.072, *p* = 0.013, Pearson’s correlation test]. Hence LPS injection abolished the correlation between C.C. and neuronal separation. ***M–O***, In an additional cohort of mice, L2/3 neurons were imaged before LPS< and again 1 and 4 d after daily LPS injections, and then after 7 d of recovery from the last LPS injection (***M***). The distributions of the C.C. (***N***) and the neuronal firing frequencies (***O***) over these time periods are shown. LPS injection caused a significant decrease in C.C., which was evident by the first day of injection and did not recover following a week after the last LPS injection. (***N***). From 4 d after LPS, the firing frequency in this mouse cohort decreased, and there was a further decrease by one week after the last LPS injection [***O***; ***N***: *F*_(3,19984)_ = 189.29, *p* = 0.002 (day 1 pre; 0.089 ± 0.004 vs day 1 post; 0.074 ± 0.003), *p* = 3.77 × 10^−9^ (day 1 pre; 0.089 ± 0.004 vs day 4; 0.035 ± 0.002), *p* = 3.77 × 10^−9^ (day 1 pre; 0.089 ± 0.004 vs day 11; 0.023 ± 0.001), *p* = 3.77 × 10^−9^ (day 1 post; 0.074 ± 0.003 vs day 4; 0.035 ± 0.002), *p* = 3.77 × 10^−9^ (day 1 post; 0.074 ± 0.003 vs day 11; 0.023 ± 0.001), *p* = 1.93 × 10^−9^ (day 4; 0.035 ± 0.002 vs day 11; 0.023 ± 0.001), one-way ANOVA followed by Tukey’s test; ***O***: day 1 pre (*n* = 320 neurons, seven fields from four mice); 0.122 ± 0.002 Hz, day 1 post (*n* = 325 neurons, seven fields from four mice); 0.122 ± 0.002 Hz, day 4 (*n* = 449 neurons, seven fields from four mice); 0.112 ± 0.002 Hz, day 11 (*n* = 737 neurons, seven fields from four mice); 0.102 ± 0.001 Hz, *F*_(3,1827)_ = 37.13, *p* = 1.00 (day 1 pre vs day 1 post), *p* = 2.43 × 10^−4^ (day 1 pre vs day 4), *p* = 3.77 × 10^−9^ (day 1 pre vs day 11), *p* = 1.54 × 10^−4^ (day 1 post vs day 4), *p* = 3.77 × 10^−9^ (day 1 post vs day 11), *p* = 5.09 × 10^−5^ (day 4 post vs day 11), one-way ANOVA followed by Tukey’s test]. Data in ***B*** shows the mean ± SEM, the dark lines in panels ***G***, ***H***, ***J***, ***K***, ***N***, ***O*** represent the means; *p < 0.05, **p < 0.01, ***p < 0.001, n.s.: non-significant.

**Figure 5.**
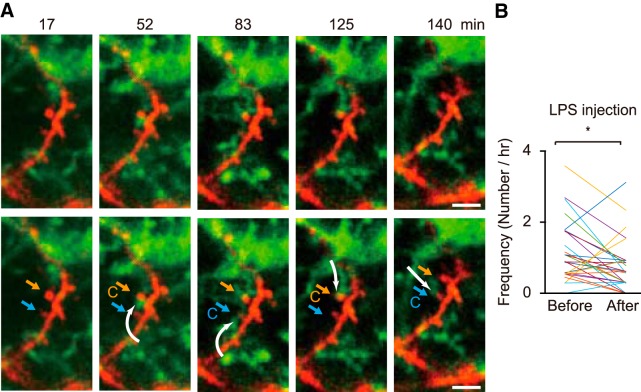
Specific activated microglial processes contact different spines, while a single spine may be contacted by different activated microglia. ***A***, As in Figure 2 for physiologic microglia, the processes of the same specific activated microglia contact two different spines (orange arrow spine at 52 min, blue arrow spine at 83 min). The same two spines are later contacted by processes originating from a different microglia. ***B***, The mean frequency of contacts between microglial processes and spines in mice with an acute injection of LPS. LPS decreases the contact frequency from 1.17 ± 0.16/h under control to 0.76 ± 0.13/h after LPS injection (*n* = 31 spines from four mice, *p* = 0.0132, paired *t* test). *p < 0.05.

We next hypothesized that only surveying, physiologic microglia have the ability to effect neural activity by microglial process contact. To test this we used daily systemic injection of LPS, a treatment know to activate microglia (Chen et al., 2014). We confirmed that systemic LPS injections for 4 d resulted in the expected morphologic changes (Fig. 4*A*,*B*) whereas control systemic saline injections did not affect microglial properties (saline-injected mice soma diameter: 40.1 ± 9.2 μm^2^, *n* = 58 cells from three mice, LPS-injected mice; 49.2 ± 11 μm^2^, *n* = 80 cells from three mice, *p* = 4.30 × 10^−7^, unpaired *t* test). In mice with LPS injection for 4 d (LPS mice), we still observed contacts between spines and microglial processes (Fig. 5*A*). When we examined contact frequency in response to an acute single LPS injection, we saw a significant decrease, from 1.17 ± 0.16 to 0.76 ± 0.13/h after LPS injections (*n* = 31 contacts from four mice; *p* = 0.0132, paired *t* test; Fig. 5*B*). As was seen in the control mice, a single microglial process in LPS-injected mice contacted different spines, and the same spine was observed to be contacted by different microglial process (Figs. 2, 5*A* ). However in LPS mice, the microglia-spine contacts had no effect on the frequency of spine Ca^2+^ transients as compared to the basal rates before or after contact [Fig. 4*C–E*; 0.013 ± 0.003 Hz (during) vs 0.012 ± 0.003 Hz (before), *p* = 0.816, *n* = 6 fields from three mice (Fig. 4*D*); 0.013 ± 0.004 Hz (during) vs 0.014 ± 0.005 Hz (after), *p* = 0.843, *n* = 6 fields from three mice (Fig. 4*E*), paired *t* test]. We again imaged Ca^2+^ transients across multiple neurons in L2/3 of the primary motor cortex (Fig. 4*F*) and plotted the C.C. against the distance between each of the neurons within the pair, as done above with the Dox on and Dox off mice (Fig. 4*G–L*). In the 4 and 9 d saline-injected control mice, there was a negative slope correlation between neuronal separation distance and C.C., as expected, indicating that neurons which were located close to each other were more likely to fire in synchrony [control (4 d injection); *r* = –0.135, *p* = 4.45 × 10^−47^ (Fig. 4*I*); control (9 d injection); *r* = –0.303, *p* = 5.61 × 10^−44^ (Fig. 4*L*), Pearson’s correlation test]. We compared these values for 4 and 9 d saline-injected mice with those of 4 and 9 d LPS-injected mice. LPS injection, for either 4 or 9 d, significantly reduced the C.C. and decreased the extent of the relationship between neuronal separation and C.C. [Fig. 4*G*,*I*,*J*,*L*; saline-injected mice (4 d injection, *n* = 9 mice); 0.12 ± 0.004, LPS-injected mice (4 d injection, *n* = 9 mice); 0.043 ± 0.002, *p* = 7.02 × 10^−72^ (Fig. 4*G*); LPS (4 d injection); *r* = –0.0681, *p* = 1.31 × 10^−21^ (Fig. 4*I*); saline-injected mice (9 d injection, *n* = 5 mice); 0.34 ± 0.008, LPS-injected mice (9 d injection, *n* = 5 mice); 0.18 ± 0.009, *p* = 3.61 × 10^−30^, Wilcoxon rank sum test (Fig. 4*J*); LPS (9 d injection); *r* = –0.072, *p* = 0.013, Pearson’s correlation test (Fig. 4*L*)]. In contrast, LPS treatment did not significantly impact on neuronal firing frequency [Fig. 4*H*,*K*; control (4 d, *n* = 464 from nine mice); 0.098 ± 0.002 Hz, LPS (4 d, *n* = 568 from nine mice); 0.098 ± 0.002 Hz, *p* = 0.841 (Fig. 4*H*); control (9 d, *n* = 141 from five mice); 0.033 ± 0.002 Hz, LPS (9 d, *n* = 108 from five mice); 0.037 ± 0.003 Hz, *p* = 0.60 (Fig. 4*K*), Wilcoxon rank sum test]. Hence the synchronized firing of neurons, and particularly for closely located neurons, is abolished by LPS-induced microglial activation.

We further investigated the time course and recovery profile for the effects of LPS injections, using four mice which received LPS injections over 4 d with repeated imaging, with the final imaging session 7 d after stopping LPS injections (Fig. 4*M–O*). Even after a single LPS injection, there was a significant decrease in the C.C., and this further decreased after 4 d of LPS injections. Following one week of recovery, the C.C. was still significantly decreased [Fig. 4*N*; *F*_(3,19984)_ = 189.29, *p* = 0.002 (day 1 pre; 0.089 ± 0.004 vs day 1 post; 0.074 ± 0.003), *p* = 3.77 × 10^−9^ (day 1 pre; 0.089 ± 0.004 vs day 4; 0.035 ± 0.002), *p* = 3.77 × 10^−9^ (day 1 pre; 0.089 ± 0.004 vs day 11; 0.023 ± 0.001), *p* = 3.77 × 10^−9^ (day 1 post; 0.074 ± 0.003 vs day 4; 0.035 ± 0.002), *p* = 3.77 × 10^−9^ (day 1 post; 0.074 ± 0.003 vs day 11; 0.023 ± 0.001), *p* = 1.93 × 10^−9^ (day 4; 0.035 ± 0.002 vs day 11; 0.023 ± 0.001), one-way ANOVA followed by Tukey’s test]. This lack of recovery of C.C. is consistent with the persistent (>28 d) activation of microglia induced by a single LPS injection (Kondo et al., 2011). The neuronal firing frequency did not change after a single LPS injection. However, in contrast to what we had observed with the 4 and 9 d treatment cohorts above, the firing frequency continued to decrease slightly over each of the subsequent imaging days [Fig. 4*O*; day 1 pre (*n* = 320 neurons, seven fields from four mice); 0.122 ± 0.002 Hz, day 1 post (*n* = 325 neurons, seven fields from four mice); 0.122 ± 0.002 Hz, day 4 (*n* = 449 neurons, seven fields from four mice); 0.112 ± 0.002 Hz, day 11 (*n* = 737 neurons, seven fields from four mice); 0.102 ± 0.001 Hz, *F*_(3,1827)_ = 37.13, *p* = 1.00 (day 1 pre vs day 1 post), *p* = 2.43 × 10^−4^ (day 1 pre vs day 4), *p* = 3.77 × 10^−9^ (day 1 pre vs day 11), *p* = 1.54 × 10^−4^ (day 1 post vs day 4), *p* = 3.77 × 10^−9^ (day 1 post vs day 11), *p* = 5.09 × 10^−5^ (day 4 post vs day 11), one-way ANOVA followed by Tukey’s test].

## Discussion

Microglial undertake continuous surveillance of neurons in healthy and diseased brain (Nimmerjahn et al., 2005; Wake et al., 2009). These microglia-neuron contacts can help sculpt the structural elements of neural circuits through formation (Parkhurst et al., 2013; Miyamoto et al., 2016) and elimination (Schafer et al., 2012) of synapses. We show here that the brief contacts between microglial processes and synapses in awake, healthy adult mice result in an acute and highly localized functional change in neural activity. Contact was followed by more Ca^2+^ transients in dendritic spines, reflecting enhanced synaptic activity. Such effects were only seen for physiologic microglia: microglia activated by LPS injections still contacted spines but did not impart on them increases in activity. Furthermore, the extent to which activity in spatially close L2/3 neurons was synchronized was also reduced following microglia activation by LPS, or following partial microglial ablation. We propose therefore that the functional consequences of the local increase in activity imparted by contact with processes of physiologic microglia is to enhance microscopic synchronization.

The LPS induced reduction in synchrony was evident after a single LPS injection, and further decreased when monitored after 4 d LPS injection. A single LPS injection (0.5 mg/kg, i.p.) has been shown to cause a sustained activation of microglia (for at least 28 d; Kondo et al., 2011) and the same 4 d LPS injection protocol (1 mg/kg, i.p.) causes a marked activation of microglia (Chen et al., 2014). The inhibition of synchronous firing of neurons was still reduced (and even more strongly inhibited) at 7 d following the last LPS injection, suggesting that sustained microglia activation may continue to inhibit neuronal synchronicity. Alternatively, a lack of microglia-induced synchronization may have a prolonged effect, as even when microglial numbers had returned to control levels following a transient ablation (in the Dox-off mice) the extent of synchrony only partially recovered. It is not known, however, whether microglia repopulating the brain following an ablation return immediately to their original physiologic phenotype. Chen et al., reported that sustained LPS-induced microglia activation was associated with “synaptic stripping” of inhibitory terminals on Layer III and V neurons in the motor cortex, that was associated with enhanced gamma band power in local field potential recordings (Chen et al., 2014). Our direct Ca^2+^ imaging revealed no increases in activity levels, if anything these tended to decrease after LPS injections or microglial ablation. Clearly, brain inflammation can impart effects on neuronal activity and cognitive processing via two broad mechanistic pathways: effects arising from a loss of physiologic microglia induced neuronal synchrony, and effects arising from changes in synapses and circuits due to consequences of activated microglia.

Coordinated neuronal activity is a fundamental component of brain activity which may arise from underlying oscillations or from temporally related sensory or motor responses. At the macroscopic level, neurons within cortical columns respond with some synchrony during specific sensory or motor modalities. However, at a more microscopic level, *in vivo* imaging of cortical activity has also demonstrated localized patterns of synchronous neural activity. Subsets of motor cortical L2/3 neurons within close (≈<100 µm) proximity, for example, respond synchronously during running, grooming or lever pulling (Dombeck et al., 2009; Hira et al., 2013). Shared excitatory cortical afferent inputs (Yoshimura et al., 2005) and/or synaptic interconnections between closely co-localized neuron pairs (Ko et al., 2011) may contribute to such microscopic L2/3 neuron synchronicity. In addition to these “wiring” mechanisms, we suggest that physiologic microglia can also help entrain pyramidal neurons into a microscopic or local synchronized circuit. The restriction of synchronization to close proximity aligns with the surveillance domain of the processes of a single microglia (<50 µm; Nimmerjahn et al., 2005). We propose that subsets of synapses within the domains of each microglia are synchronized. How these processes know which neurons are to be recruited into a neural circuit associated with a particular modality is unclear, but neuronal activity can attract microglial contact (Li et al., 2012). It may be that neurons participating most strongly in a particular response preferentially attract microglial process, which then further enhance the activity of these neurons and thereby help synchronize these neurons into a circuit or engram.

The functional implications of microglial-nerve contacts in synchronizing local circuits we report here were derived from male mice. A number of gender differences in microglial dynamics and responses have been reported. As reviewed by Rahimian et al. (2018), the morphology and phagocytic phenotypes of male and female microglia differ over development, and gender differences in the inflammatory response to ischemia have also been observed in adult brain. Whether gender differences in the physiologic microglia-neuron interactions we report here for adults also exist is unclear, but will be of interest to consider in light of the gender differences in inflammation-associated risk factors for schizophrenia and in the incidence of autism spectrum disorders (Lenz and McCarthy, 2015).

Our result has potential implications for understanding how immune system activation may impact on cognitive function. Maternal infection and chronic inflammatory disorders in adults (e.g., periodontitis) are associated with an increase incidence of brain disorders characterized by cognitive impairments (Noble et al., 2009; Knuesel et al., 2014), while some genetic brain disorders have microglial genes as their proposed basis (see Wake et al., 2013). We have previously speculated that microglial activation and/or dysfunction may be causative for cognitive disorders (Wake et al., 2013), and our current results suggests an additional putative mechanistic link for such an association. Further understanding of the signaling pathways and behavioral consequences of this enhancement of synaptic activity by physiologic microglial surveillance may provide insights to address cognitive disorders involving altered immune and microglial status.

## Synthesis

Reviewing Editor: Maiken Nedergaard, University of Rochester Medical Center Decisions are customarily a result of the Reviewing Editor and the peer reviewers coming together and discussing their recommendations until a consensus is reached. When revisions are invited, a fact-based synthesis statement explaining their decision and outlining what is needed to prepare a revision will be listed below. The following reviewer(s) agreed to reveal their identity: Marie-Ève Tremblay, Frank Kirchhoff.

## Author Response

Specific Responses to the Reviewers Comments

Editorial Synthesis of reviews:

“(1) discussion of gender issues.(2) Expand the discussion regarding microglial/synapse/neuronal interactions including the lack of effect of doc. (3) Discuss the lack of longterm recovery, and worsening of synchrony.(4) Address all of the reviewers concerns listed in their comments.

The reviewers' comments are:

Reviewer #1:

The data within the manuscript remain preliminary and largely phenomenological. While the authors adequately responded to several of our original requests, there remain significant unvalidated experiments to support their claims. Unfortunately the experiments performed between the first and second submission did not address our concerns about whether or not neuronal synchrony recovers after microglial ablation or perturbation as the authors did not verify that normal microglial surveillance resumed after either of their paradigms. Specific comments are outlined below:

Specific responses are listed below.

1. The authors did not provide adequate justification within the text (methods or discussion) or within their response to our request for the use of female mice in experiments to overlook the use of only male mice in these experiments.

As indicated in our previous response, we did not feel it feasible to repeat our experiments in females as we are reporting a new aspect to the functional consequences of microglia-neuron interactions and not investigating gender differences in microglia-neuron interactions. We hence used only male mice to specifically exclude variations due to changes in menstrual cycle. We are aware of reported gender differences in the microglia field, but further felt that to discuss them would be just superficial as we could not shed any new information on how they would contribute to our specific phenomena. However, we have now conceded to point out that we have only used males, that gender differences exist and to indicate that further studies to pursue whether gender differences are warranted. We have added a specific paragraph to the discussion (p25, line3) about gender differences in microglial responses.

2. I believe Figure 3 legend is mislabeled and implies microglia are EGFP+ in the Iba1-tTa::tetO-DTA mice. Are these microglia in fact labeled with anti-Iba1 immunostaining? This needs to be much clearer in the text.

We thank the reviewer for pointing this out. Fig 3A is indeed immunohistochemistry, and we have now corrected the legend and clarified this

3. The authors performed a dox-recovery experiment to address long-lasting neuronal synchrony in response to a short-term microglial ablation. However, they do not quantify the microglial recovery. Is 42 days sufficient to repopulate microglia/do the microglia tile the brain normally within this time period?

The lack of investigation into microglial/synaptic interactions within the dox treated animals is disappointing.

We have now gone back and counted microglia density in a cohort of mice at one week after reintroduction of Dox. The density had completely recovered after 1 week of re-introducing Dox and we conclude at 42 days microglia density is also back to control. The sample (Fig 3A) and quantified data (Fig 3B) showing return of microglia density is now shown in revised Figure 3.

We have now included an additional paragraph in the discussion (p22, line 15-p23, line17) about the long-term effects of inhibiting physiological microglia, that address the lack of complete recovery of synchrony when Dox was returned, and at 1 week following the cessation of LPS injections.

4. We requested the authors perform chronic imaging on LPS injected animals, several weeks after the animals had fully recovered from administration. While we appreciate the experiments showing that 7 days post LPS administration there remain neurological consequences, the authors did not perform chronic imaging on these animals as requested. 1 week of recovery is insufficient as referenced by us in our original review of the manuscript as well as by Kondo et al. Kondo et al also used a single bolus of 0.5 mg/kg LPS, and the authors did not respond directly to our concern that daily injections of 1mg/kg LPS were very high.

We have now specifically referred to the single 0.5 mg/kg LPS dose used by Kondo et al (2011; resulting in microglial activation for at least 28 days) and the 4-daily 1mg/kg injection protocol by Chen et al (2014) - both protocols lead to microglia activation. This is part of our new paragraph about lack of Dox effects/full recovery (p22, line 15-p23, line17). Our 1mg/kg LPS dose regime (1/day; four days) was based exactly on the dose regime of Chen et al., (Nature Comms, 2014) as this was shown to activate microglia as measured by morphological parameters and by microglia inhibitory synapse ‘stripping’. The key point of our study was to activate microglia, not to tease out the sensitivity or dose relationship.

In regards to the more chronic imaging, we did image at 1 week after stopping LPS. It's difficult technically to image chronically over longer periods due to issues related to stability of AAV mediated fluorophore levels (amongst other challenges). Our microglia ablation provides some insights into whether the synchronization can recover. Further experiments to look at long-term impacts of a transient activation or depletion of microglia would, we agree, be interesting but we believe reporting and characterizing the synchronization effect is novel and sufficient.

5. We are surprised the authors did not comment on the new findings from their increased data collection in response to our original suggestions.

a. Reintroducing dox into the animals' diets appears to have had no recovery of paired neuronal firing. While the authors did not sufficiently investigate if this period of recovery was sufficient to repopulate microglia, this is nevertheless data worth discussing.

As discussed for point 3 above, we have now counted microglia after returning Dox to the diet, showing that microglia density returns to control levels after 1 week. Reintroducing Dox appears to have a partial recovery for the synchronization of paired neurons, although this is not complete. We have added new discussion about why the lack of synchrony may be sustained beyond this 1 week in a new paragraph in the Discussion on p22, line 15-p23, line17.

b. Although 1 week of recovery from LPS injection is known to be insufficient for brain recovery, it seems the change in synchrony remains (and even worsens) days after the last administration.

As stated above, we have extended our discussion of the recovery time course, for both the LPS and the Dox-re-on conditions. This includes pointing out the apparent worsening of synchrony straight after LPS. (p22, line 15-p23, line17).

Reviewer #2

The authors provide compelling evidence that microglial contacts are synchronized with neuronal network activity in healthy mice. This is an important observation for our knowledge on the mechanisms of neurotransmission in cell ensembles.

We appreciate for the reviewer for their nice comments.
